# Does timing matter? The role of health information shocks in measuring willingness to pay

**DOI:** 10.1007/s10198-025-01774-7

**Published:** 2025-04-17

**Authors:** Carolin Brinkmann, Sebastian Neumann-Böhme, Werner B. F. Brouwer, Tom Stargardt

**Affiliations:** 1 Hamburg Center for Health Economics, University of Hamburg, Hamburg, Germany; 2 Erasmus School of Health Policy and Management, Erasmus University Rotterdam, Rotterdam, The Netherlands

**Keywords:** Willingness to pay, Information shock, Health shock, Vaccination, I10, I12

## Abstract

**Objectives:**

The *optimal point in time* to measure willingness-to-pay (WTP) remains unclear. We investigated the role of health information shocks (HIS) in individuals’ WTP, analyzing the extent to which news of SARS-CoV-2 infections among people they know/themselves altered WTP for booster vaccinations.

**Methods:**

We elicited WTP in eight European countries using the European Covid Survey. First, we presented participants with a hypothetical setting recommending a booster vaccination that had to be paid out-of-pocket. To measure WTP, we elicited a lower and upper WTP limit, and a WTP value contingent on both of these. To measure HIS, we asked about the duration since participants received news of COVID-19 cases among people they know (including themselves), as well as the degree of personal connection to these cases and their severity. We used a two-part model to estimate the association between HIS and individuals’ WTP.

**Results:**

Among the 5809 observations, 76.8% stated a WTP for a booster vaccination greater than €0. At least one HIS was reported by 61.9% of participants. The occurrence of a HIS was associated with an increase in WTP of €14.54 (logistic: *P* <.0001, gamma: *P* =.1493) compared to no HIS. The WTP was higher when the HIS occurred in the four weeks before the survey. Controlling for socio-demographic and COVID-19 covariates decreased significance and effect sizes.

**Conclusion:**

Our findings suggest that a recent HIS is associated with a higher probability of having a positive WTP. Timing, in relation to some relevant event, therefore may matter when measuring WTP for health interventions. If so, finding the optimal point in time to measure WTP is difficult and may depend on the policy question under consideration.

**Supplementary Information:**

The online version contains supplementary material available at 10.1007/s10198-025-01774-7.

## Introduction

To quantify the value of non-marketed goods and services such as health care interventions, health economic evaluations often seek to elicit the maximum amount that individuals are willing to pay for them. This amount can then be used to estimate the value of the interventions in monetary terms, facilitating comparisons between them. In this way, willingness-to-pay (WTP) can help policymakers assess value for money and prioritize different interventions based on individual preferences as part of their allocative decisions [1–5].

In healthcare, this approach is especially important because the absence of market prices and the desire of policymakers to align their decisions with the preferences of citizens mean that value is usually not observable. WTP therefore has become an established measure in preventive and curative health interventions [6, 7]. Yet despite their broad use and theoretical underpinnings, the validity of WTP estimates has been criticised, for example due to their insensitivity to scale [8] and hypothetical bias [9]. Additionally, WTP values are known to be associated with the elicitation procedure, framing, and study design [10–18].

Another issue to consider when designing WTP exercises in the context of health interventions is *when* to ask participants for a valuation. Just as the phenomenon that adaptation may affect subjective health-related quality of life [19–22], the timing of a WTP exercise in relation to the occurrence of some relevant ‘shock’ may be associated with its results. This may especially be the case if participants have experienced a health event or health information shock. The concept of information shocks has been the subject of recent health research [23–27]. In the present study, we aimed to contribute to this literature by exploring the role of health information shocks (HIS) in relation to WTP. To do so, we focused on the example of SARS-CoV-2 booster vaccinations, eliciting the hypothetical WTP for these in eight European countries and investigating the extent to which WTP was associated with the timing of a HIS. We defined a HIS either as having experienced a COVID-19 infection oneself or having heard of infections among people one knows. At the time of data collection in December 2021 and January 2022, total cumulative COVID-19 cases were between 8,180.7 (Germany) and 22,777.1 per 100,000 persons (United Kingdom) in Europe [28], i.e., a majority of the population had not experienced a COVID-19 infection (yet). While the Delta variant was predominant in incident cases in late December 2021, the more contagious omicron variant only started to spread in Europe in early 2022 [29].

Our results suggest that the occurrence of a HIS is associated with WTP estimates, especially in the short run. A HIS might prompt individuals to be willing to pay for the offered good. The observed association suggests that the timing of WTP surveys might influence their results and ultimately affect allocation decisions.

## Methods

### The European Covid Survey

The European Covid Survey (ECOS) was an online survey that elicited information on the attitudes, behavior, worries, and health characteristics of European residents aged 18 years or older approximately every two months between April 2020 and December 2022. The samples consisted of approximately 1000 participants per country and wave and were representative of the general population in each country in terms of gender and age. The participating countries were Denmark, France, Germany, Italy, Portugal, the Netherlands, the United Kingdom (UK), and, after July 2021, Spain. Participants were recruited by the market research company Dynata. Participation was anonymous and required written informed consent. The questionnaires were developed in English, translated into the various national languages, and piloted in 10% of each sample. Further information can be found elsewhere [30–33]. The present analysis is based on the 9th wave of ECOS data, collected between 23 December 2021 and 11 January 2022.

### Measuring health information shocks (HIS)

We defined health information shocks (HIS) as important new information received by participants about their own health or the health of someone in their social environment. Based on this definition, we measured HIS by asking participants about (a) the duration since they heard news of COVID-19 cases among people they know (including themselves), (b) their degree of personal connection to these cases, and (c) the severity of these cases.

To do so, we used a matrix in which participants could tick boxes for each combination of temporal (when? ) and relational (who?) information (see Online Resource). The duration since receiving news about a case of COVID-19 was shown using two-week intervals ranging from “0 to 2 weeks” to “7 to 8 weeks”, and complemented by an open interval of “more than 8 weeks”. The degree of personal connection to these cases was shown as “household”, “family”, “friends”, “colleagues”, and “no” or “don’t know”. Self-shocks (infections among participants themselves) were implicitly included in the category “household”. Lastly, participants were asked to indicate the overall severity of the majority of the COVID-19 cases, with the options ranging from “more severe than expected” to “milder than expected”, and including a mixed category (“mixed, i.e., half of the cases more severe than expected, and half of the cases milder than expected”).

When analyzing the data, we treated the category “Don’t know” the same as the category “No” based on the assumption that a case of COVID-19 that the participant did not remember or did not consciously perceive would be the same as that of the absence of a case of COVID-19.

### WTP measurement

To measure WTP, we presented participants with a hypothetical scenario in which (a) a booster vaccination was recommended, (b) it had to be paid out of pocket and the costs would not be reimbursed by health insurance or the government, (c) participants’ health and vaccination status permitted a booster vaccination, (d) participants could choose their preferred COVID-19 vaccine brand, and (e) the vaccination would be administered in a convenient location.

Similar to Himmler et al. [34], we opted for a guided measurement procedure to help participants form their WTP for a non-marketed good. An initial filter question identified whether individuals were willing to pay a positive amount for a booster vaccination. For those with a positive amount, we followed a three-step approach to elicit the WTP for a booster shot. First, we asked for the amount participants would certainly pay, using a scale ranging from €0 to €150 with visual anchoring points every €30. Alternatively, an open-ended question allowed participants to indicate a WTP greater than €150. Second, we asked for the amount participants would be unwilling to go beyond, using the same instruments as in the first step. Lastly, participants were asked to state their WTP using an open-ended question phrased in such a way that it reminded them of, and was conditional on, the previously set interval (see Online Resource).

For participants who stated in the initial filter question that they had no WTP or a WTP of €0 in the third step, we asked for their motivation in order to differentiate between true zeros and protest answers that would indicate a violation of the hypothetical setting. Motivation options included (1) not needing the booster shot because the participant believed he or she would not become ill with COVID-19, (2) not being able to afford the booster shot, (3) the booster shot being of no value to the participant because of worries about potential side effects, (4) vaccines generally having no value to the participant, (5) not wanting to pay because of the belief that vaccines should be paid by the government, and (6) other reasons. Options 1 to 4 were considered to be reflective of a true WTP of €0. Options 5 and 6 were regarded as protest answers and thus excluded from the analysis. We excluded extreme values, defined as WTP values above the adjusted gross disposable income of households per capita for the year 2020 for each country [35].

We asked for valuations in participants’ local currency. For our analyses, we converted pound sterling and Danish krone to euros using the exchange rate of the European Central Bank from 23 December 2021. We adjusted WTP values for purchasing power parity based on the 2020 Eurostat purchasing power adjusted gross domestic product per capita [36]. We used conditional pathways, dynamic validation, and piped text to ensure the quality and consistency of answers. Lastly, we identified careless responders– e.g., those who completed the questionnaire in less than a third of the median survey duration per country (so-called speeders)– and dropped these observations [37].

### Statistical analysis

We calculated descriptive statistics for the sample, as well as WTP and HIS values. All analyses were based on the WTP response to the last elicitation step. We estimated the difference in WTP depending on the occurrence of HIS using a two-part model because of the continuous, non-negative nature of the dependent variable WTP [38] and a large number of zeros. In the first part, we modeled the probability of the WTP being positive with logistic regression (WTP > 0; WTP = 0). In the second part, we estimated the WTP, conditional on it being positive, using a gamma distribution and log-link function.

We considered four different model specifications for HIS: Model 1 included a binary variable indicating whether a participant had been subject to a HIS. Model 2 included a cardinal measurement of HIS intensity (i.e., the number of HIS experienced and its quadratic term). Model 3 referred to the most recent HIS experienced by a participant; it included the duration since the most recent HIS and the severity of the majority of COVID-19 cases. Lastly, Model 4 combined the specifications of Models 2 and 3 by adding the total number of HIS experienced in order to control for experience with COVID-19. We chose a linear rather than a squared specification for the number of HIS experienced by a participant because the results of Model 2 indicated that the tipping point was approximately six times the maximum reported number of HIS.

Additionally, Model 4 controlled for the following predictors of WTP reported in the literature: socio-demographic characteristics, perceived threat from the disease, perceived benefits, and prior knowledge of the health intervention [39]. We operationalized socio-economic status using a binary variable for gender, six categories for age, and a three-level education variable based on each country’s educational qualifications (see Online Resource), as well as a four-category variable as a proxy for income indicating the extent to which participants were “able to make ends meet”, ranging from “easily” to “with great difficulty”. We operationalized the perceived threat from the disease as quality of life measured using EQ-5D-5 L and as subjective risk to own health from COVID-19 ranging from “no risk at all” to “very high risk”. In turn, we operationalized the perceived benefit of the health intervention by including (a) the vaccination status of the participant, measured using five levels ranging from not being vaccinated/not yet being vaccinated to having received up to three vaccination shots, and (b) the vaccination status of peers in four levels (“none”, “just a few”, “about half”, “most”). In addition, we included country fixed-effects and a risk-aversion measure based on Barsky et al. [40] with four levels ranging from “very low” to “high”. We chose this measure based on the assumption that it was independent of the COVID-19 pandemic and would be unaffected by the HIS examined in our study.

We calculated average marginal effects (AME) to facilitate interpretation of the results of the two-part model in one estimate. We conducted the analysis using the “twopm” command [41] in Stata 17.

## Results

### Descriptive results

**Fig. 1:**
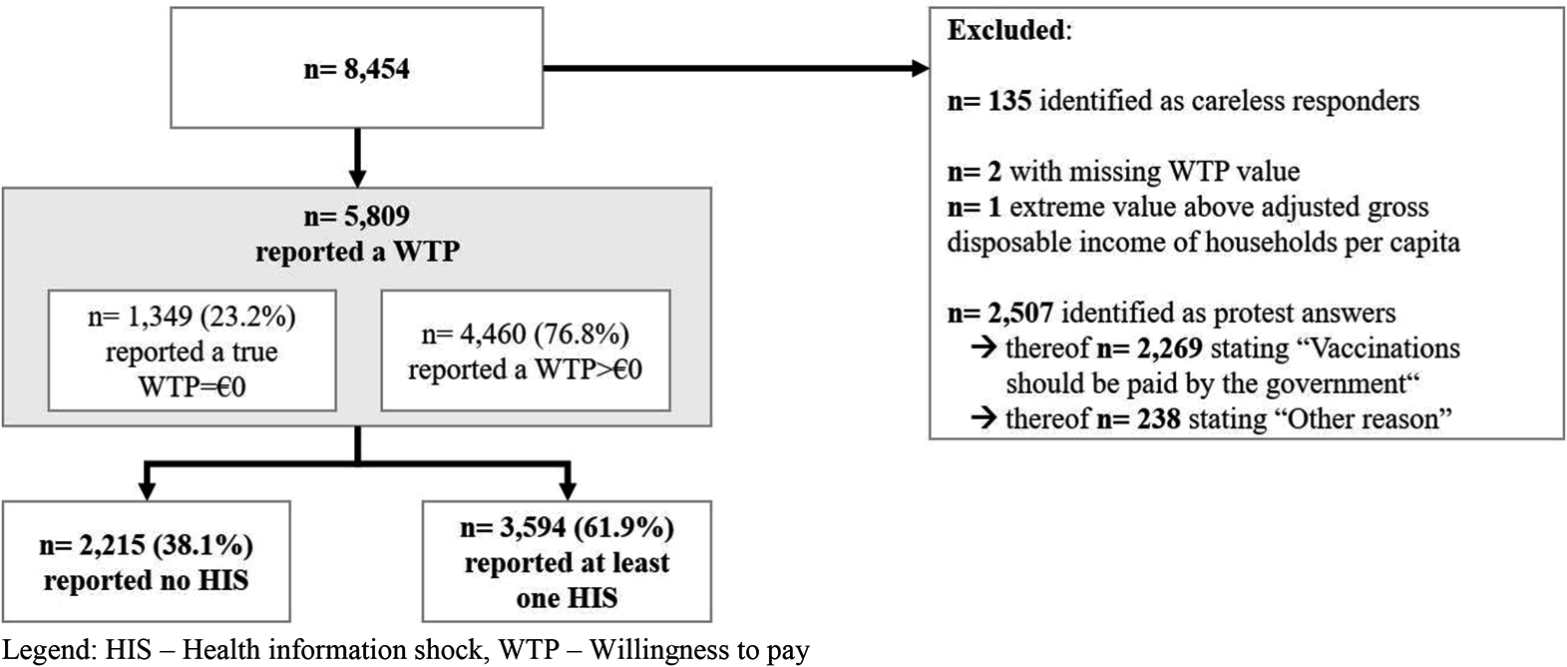
Flowchart of the sample. HIS– Health information shock, WTP– Willingness to pay

After we excluded careless responders, extreme values, protesters, and missing information, the final sample comprised 5809 observations (Fig. 1). Detailed sample characteristics can be found in Table 1.

**Table 1:** Sample characteristics

	Sample characteristics per HIS status (*N* = 5809)				Sample characteristics per WTP statement (*N* = 5809)			
	No HIS (*n* = 2215)		At least one HIS (*n* = 3594)		WTP = 0 (*n* = 1349)		WTP > 0 (*n* = 4460)	
	n	%	n	%	n	%	n	%
Gender								
Female	1132	51.11	1899	52.84	804	59.60	2227	49.93
Male	1083	48.89	1695	47.16	545	40.40	2233	50.07
Age (years)								
18–24	107	4.83	325	9.04	118	8.75	314	7.04
25–34	267	12.05	657	18.28	263	19.50	661	14.82
35–44	351	15.85	765	21.29	314	23.28	802	17.98
45–54	417	18.83	644	17.92	299	22.16	762	17.09
55–64	407	18.37	577	16.05	187	13.86	797	17.87
65 or older	666	30.07	626	17.42	168	12.45	1124	25.20
Education level								
High	867	39.14	1982	55.15	527	39.07	2322	52.06
Middle	944	42.62	1197	33.31	587	43.51	1554	34.84
Low	404	18.24	415	11.55	235	17.42	584	13.09
Ability to make ends meet								
Easily	350	15.80	570	15.86	115	8.52	805	18.05
Fairly easy	893	40.32	1543	42.93	418	30.99	2018	45.25
With some difficulty	780	35.21	1206	33.56	602	44.63	1384	31.03
With great difficulty	192	8.67	275	7.65	214	15.86	253	5.67
Country								
Denmark	223	10.07	542	15.08	161	11.93	604	13.54
France	409	18.47	421	11.71	250	18.53	580	13.00
Germany	388	17.52	308	8.57	173	12.82	523	11.73
Italy	218	9.84	470	13.08	147	10.90	541	12.13
Netherlands	254	11.47	414	11.52	213	15.79	455	10.20
Portugal	217	9.80	486	13.52	84	6.23	619	13.88
Spain	170	7.67	506	14.08	154	11.42	522	11.70
United Kingdom	336	15.17	447	12.44	167	12.38	616	13.81
Risk aversion								
Very low	684	20.66	1459	29.14	308	22.83	1312	29.42
Low	373	11.27	689	13.76	158	11.71	553	12.40
Moderate	261	7.88	555	11.08	130	9.64	459	10.29
High	1993	60.19	2304	46.02	753	55.82	2136	47.89
Vaccination status								
No	334	15.08	242	6.73	529	39.21	47	1.05
Not yet, but I intend to	35	1.58	61	1.70	55	4.08	41	0.92
Yes, the first shot	47	2.12	157	4.37	48	3.56	156	3.50
Yes, both shots	654	29.53	1432	39.84	459	34.03	1627	36.48
Yes, three shots (booster)	1145	51.69	1702	47.36	258	19.13	2589	58.05
Peers’ vaccination status								
Most	1731	78.15	2811	78.21	764	56.63	3778	84.71
About half	166	7.49	300	8.35	202	14.97	264	5.92
Just a few	211	9.53	417	11.60	271	20.09	357	8.00
None	107	4.83	66	1.84	112	8.30	61	1.37

In total, 23.2% of participants expressed a WTP of €0, whereas 76.8% expressed a WTP greater than €0. Moreover, 61.9% of participants reported experiencing a HIS (i.e., at least one COVID-19 case), whereas 38.1% reported not experiencing any HIS. Of those who reported experiencing a HIS, 29.4% reported one, 22.0% reported two, 12.4% reported three, 10.6% reported four, and 14.2% reported five. The remaining 11.4% reported between six and the maximum number of 20 HIS. A large majority of participants who reported experiencing a HIS indicated that their most recent one had occurred zero to two weeks before the survey (69.5% of those who experienced a HIS), whereas 11.5% indicated that it had occurred three to four weeks and 9.9% that it had occurred more than eight weeks before the survey.

On average, participants were willing to pay €48.58 for a booster vaccination. The WTP ranged from €0.00 to €7542.60. Participants who reported experiencing at least one HIS indicated having a higher WTP on average than those who reported not experiencing a HIS. The difference in the mean WTP between those with and those without a HIS varied across countries. With regard to the temporal proximity of a HIS, the mean WTP was €55.25 among individuals whose most recent HIS occurred in the two weeks before the survey and €60.62 among individuals whose most recent HIS occurred in the three to four weeks before the survey. Among individuals whose most recent HIS occurred more than four weeks before the survey, the WTP was lower than that among participants with a more recent HIS (5–6 weeks: €44.81, 7–8 weeks: €43.64, > 8 weeks: €47.57, Table 2).

**Table 2:** Mean WTP per country and HIS status

	**HIS (n = 3594)**			No HIS (n = 2215)				Overall (N = 5809)		
	Mean (in €)	SD (in €)	Median (in €)	Mean (in €)	SD (in €)	Median (in €)	T-test HIS vs. No HIS	Mean (in €)	SD (in €)	Median (in €)
**Overall**	54.12	144.33	35.56	39.58	144.10	19.81	.0002	48.58	144.4	29.40
Country										
Denmark (n = 765)	66.30	76.55	51.62	45.91	60.81	33.89	.0001	60.36	72.87	43.01
France (n = 830)	41.13	42.50	29.72	37.46	296.83	11.89	.8042	39.32	210.43	19.81
Germany (n = 696)	70.51	78.41	52.29	51.77	92.39	29.05	.0039	60.06	86.92	40.67
Italy (n = 688)	49.89	60.75	35.56	33.68	58.29	17.78	.0010	44.75	60.41	31.11
Netherlands (n = 668)	74.92	376.42	37.71	28.50	35.45	18.86	.0131	57.26	297.86	31.43
Portugal (n = 703)	30.98	43.15	21.71	24.19	27.11	14.48	.0117	28.88	39.02	20.99
Spain (n = 676)	38.16	40.99	28.00	35.63	95.05	12.04	.7366	37.52	59.34	24.00
United Kingdom (n = 783)	68.72	117.19	47.04	48.06	94.21	29.40	.0064	59.85	108.35	35.28
Time since most recent HIS										
0–2 weeks	55.25	161.88	36.13							
3–4 weeks	60.62	115.27	43.01							
5–6 weeks	44.81	60.64	32.53							
7–8 weeks	43.64	42.48	31.43							
More than 8 weeks	47.57	89.62	30.11							
Degree of personal connection to COVID-19 cases										
Household	60.24	74.91	43.56							
Family	55.99	210.26	36.16							
Friends	50.02	67.63	34.54							
Colleagues	47.64	65.86	34.67							
Severity of majority of COVID-19 cases known by participant										
Milder than expected	38.27	62.62	24.00							
A bit milder than expected	50.82	70.51	34.86							
As severe/mild as expected	60.12	254.36	38.54							
A bit more severe than expected	69.30	81.93	54.22							
More severe than expected	66.74	61.50	57.90							
Mixed, i.e., half of cases more severe, half of cases milder than expected	45.74	118.50	24.44							

HIS– Health information shock, SD - Standard deviation

With regard to the degree of personal connection to COVID-19 cases, the differences in WTP among participants were small. Among those whose closest COVID-19 case was in the household, the WTP for a booster vaccination was €60.24, followed by a WTP of €55.99 if the closest case was in the family, €50.02 if it was among friends, and €47.64 if it was among colleagues. With regard to the severity of HIS, the mean WTP for those who reported that the severity of the majority of cases they heard of or experienced was milder than expected was €38.27, with those reporting higher severity also indicating a higher mean WTP (Table 2).

### Regression results

The results of Model 1 show that HIS was associated with the WTP for a booster vaccination (Table 3), with the report of any HIS being associated with an increase in WTP of €14.54 compared to no HIS. In Model 2, our findings suggest that the total number of reported HIS and the square of this number were also associated with WTP, with the squared number of HIS indicating that an increasing number of HIS was associated with a decrease in WTP, but with a tipping point at approximately six times the maximally reportable number of HIS.

**Table 3:** Regression results model 1 and model 2

	Model 1													Model 2												
	Logistic						Gamma						AME	Logistic						Gamma						AME
	Est.	SE	p value	95%CI			Est.	SE	p value	95%CI			Est.	Est.	SE	p value	95%CI			Est.	SE	p value	95%CI			Est.
Intercept	0.74	(0.05)	< 0.0001	0.65	,	0.83	4.07	(0.07)	< 0.0001	3.94	,	4.20		0.80	(0.04)	< 0.0001	0.72	,	0.88	4.01	(0.05)	< 0.0001	3.90	,	4.12	
HIS (Yes)	0.80	(0.06)	< 0.0001	0.67	,	0.92	0.12	(0.08)	0.1493	-0.04	,	0.28	14.54													
No. Of HIS														0.32	(0.03)	< 0.0001	0.26	,	0.37	0.08	(0.03)	0.0077	0.02	,	0.14	7.26
No. Of HIS²														-0.02	(0.00)	< 0.0001	-0.02	,	-0.01	0.00	(0.00)	0.1779	-0.01	,	0.00	-0.39

AME– Average marginal effect, Est.– Estimate, No.– Number, SE– Standard error

In Model 3, which considered the duration since the most recent HIS, the association of a HIS with WTP was positive and stronger if the HIS occurred in the four weeks before the survey (AME for zero to two weeks: €20.05; AME for three to four weeks: €29.20) compared to no HIS (logistic: *P* <.003 for all temporal proximity levels, gamma: *P* =.0492 for reporting having experienced a HIS three to four weeks before the survey and all other levels *P* >.05). Moreover, WTP was positively associated with the severity of HIS: compared to the WTP among those who reported the majority of cases as being as severe or as mild as expected, the WTP was lower among participants who reported the majority of COVID-19 cases as being milder (AME: €-19.81, logistic: *P* <.0001, gamma: *P* =.0248) than expected. Other levels of severity were not associated with the WTP (Table 4).

**Table 4:** Regression results model 3 and model 4

	Model 3								Model 4													
	Logistic				Gamma				AME	Logistic				Gamma				AME				
	Est.	SE	p value	95%CI			Est.	SE	p value	95%CI			Est.	Est.	SE	p value	95%CI	Est.	SE	p value	95%CI	Est.
Intercept	0.74	(0.05)	<.0001	0.65, 0.83			4.07	(0.06)	<.0001	3.94, 4.19				1.57	(0.37)	<.0001	0.84, 2.30	4.44	(0.31)	<.0001	3.83, 5.05	
No. Of HIS														0.12	(0.03)	0.0001	0.06, 0.17	0.03	(0.02)	0.0401	0.00, 0.07	2.34
Temporal proximity																						
0–2 weeks since HIS	1.04	(0.11)	<.0001	0.83, 1.25			0.19	(0.11)	0.0953	-0.03, 0.41			20.05	0.50	(0.16)	0.0025	0.17, 0.82	-0.03	(0.10)	0.8060	-0.23, 0.18	1.92
3–4 weeks since HIS	1.03	(0.16)	<.0001	0.71, 1.34			0.34	(0.17)	0.0492	0.00, 0.68			29.20	0.55	(0.21)	0.0095	0.14, 0.97	0.10	(0.13)	0.4427	-0.16, 0.36	8.95
5–6 weeks since HIS	0.86	(0.19)	<.0001	0.48, 1.24			0.07	(0.22)	0.7437	-0.35, 0.50			12.20	0.39	(0.25)	0.1221	-0.10, 0.88	-0.16	(0.16)	0.3311	-0.47, 0.16	-4.60
7–8 weeks since HIS	0.82	(0.27)	0.0022	0.29, 1.34			0.02	(0.30)	0.9359	-0.56, 0.61			9.60	0.26	(0.33)	0.4365	-0.40, 0.92	0.00	(0.22)	0.9976	-0.42, 0.43	1.80
More than 8 weeks since HIS	0.80	(0.16)	<.0001	0.48, 1.11			0.12	(0.18)	0.5106	-0.24, 0.48			14.09	0.22	(0.20)	0.2882	-0.18, 0.61	0.02	(0.13)	0.8997	-0.25, 0.28	2.30
No HIS	.						.						.	.				.				
Severity of HIS																						
Milder than expected	-0.70	(0.12)	<.0001	-0.93, -0.46			-0.31	(0.14)	0.0248	-0.57, -0.04			-19.81	-0.41	(0.16)	0.0093	-0.71, -0.10	-0.08	(0.10)	0.4451	-0.27, 0.12	-6.14
A bit milder than expected	-0.05	(0.14)	0.7283	-0.32, 0.22			-0.16	(0.14)	0.2297	-0.43, 0.10			-7.99	-0.03	(0.17)	0.8767	-0.35, 0.30	0.01	(0.10)	0.8871	-0.18, 0.21	0.53
As severe/mild as expected	.						.						.	.				.				
A bit more severe than expected	0.21	(0.16)	0.1852	-0.10, 0.53			0.12	(0.15)	0.4333	-0.17, 0.40			8.18	0.36	(0.19)	0.0589	-0.01, 0.73	0.05	(0.11)	0.6595	-0.16, 0.26	4.53
More severe than expected	0.09	(0.18)	0.6228	-0.27, 0.45			0.10	(0.17)	0.5744	-0.24, 0.44			5.98	0.39	(0.23)	0.0842	-0.05, 0.83	0.06	(0.13)	0.6625	-0.20, 0.32	5.23
Mixed, i.e. half more severe, half milder than expected	-0.42	(0.18)	0.0193	-0.78, -0.07			-0.23	(0.20)	0.2649	-0.62, 0.17			-13.84	-0.40	(0.23)	0.0798	-0.84, 0.05	-0.06	(0.15)	0.6679	-0.35, 0.23	-5.48
No HIS	.						.						.	.				.				
Female														0.20	(0.08)	0.0180	0.03, 0.36	-0.02	(0.06)	0.7070	-0.13, 0.09	0.19
Age																						
18–24 years old														-0.43	(0.19)	0.0245	-0.81, -0.06	0.22	(0.14)	0.1067	-0.05, 0.48	9.36
25–34 years old														-0.65	(0.16)	<.0001	-0.96, -0.35	0.02	(0.11)	0.8248	-0.18, 0.23	-2.63
35–44 years old														-0.64	(0.15)	<.0001	-0.93, -0.35	0.02	(0.10)	0.8023	-0.16, 0.21	-2.48
45–54 years old														-0.78	(0.14)	<.0001	-1.05, -0.50	-0.01	(0.09)	0.9420	-0.18, 0.17	-4.87
55–64 years old														-0.24	(0.15)	0.1185	-0.53, 0.06	-0.04	(0.09)	0.6790	-0.21, 0.13	-2.95
65 years old and older														.				.				
Education level																						
High														0.12	(0.13)	0.3525	-0.13, 0.37	0.15	(0.09)	0.0940	-0.03, 0.33	7.67
Middle														-0.11	(0.13)	0.3819	-0.36, 0.14	0.19	(0.09)	0.0423	0.01, 0.37	7.99
Low														.				.				
Level of making ends meet																						
Easily														1.96	(0.19)	<.0001	1.59, 2.33	0.47	(0.14)	0.0011	0.19, 0.75	37.82
Fairly easy														1.45	(0.15)	<.0001	1.17, 1.74	0.16	(0.13)	0.2140	-0.09, 0.42	16.80
With some difficulty														0.67	(0.14)	<.0001	0.40, 0.94	0.08	(0.13)	0.5511	-0.18, 0.33	8.07
With great difficulty														.				.				
Country																						
Denmark														-0.03	(0.17)	0.8505	-0.37, 0.31	-0.04	(0.11)	0.7088	-0.27, 0.18	-2.24
Spain														-0.28	(0.17)	0.0870	-0.61, 0.04	-0.43	(0.12)	0.0002	-0.66, -0.20	-19.16
France														-0.16	(0.16)	0.2942	-0.47, 0.14	-0.47	(0.11)	<.0001	-0.69, -0.24	-20.11
Italy														0.43	(0.18)	0.0143	0.09, 0.78	-0.36	(0.11)	0.0018	-0.58, -0.13	-13.40
Netherlands														-0.29	(0.16)	0.0786	-0.61, 0.03	-0.02	(0.12)	0.8371	-0.26, 0.21	-3.02
Portugal														1.12	(0.20)	<.0001	0.74, 1.51	-0.80	(0.12)	<.0001	-1.02, -0.57	-25.96
United Kingdom														-0.30	(0.17)	0.0697	-0.63, 0.02	-0.16	(0.11)	0.1528	-0.38, 0.06	-9.10
Germany														.				.				
Health Problematization Index														0.00	(0.01)	0.8937	-0.03, 0.02	0.01	(0.01)	0.3697	-0.01, 0.03	0.40
Risk to own health from COVID-19																						
No risk at all														-1.40	(0.20)	<.0001	-1.78, -1.02	-0.31	(0.15)	0.0393	-0.60, -0.01	-22.92
Little risk														-0.66	(0.16)	<.0001	-0.98, -0.34	-0.05	(0.11)	0.6249	-0.26, 0.15	-7.03
Moderate risk														-0.31	(0.15)	0.0397	-0.61, -0.01	-0.14	(0.09)	0.1313	-0.33, 0.04	-9.13
High risk														-0.17	(0.16)	0.2864	-0.49, 0.15	-0.04	(0.10)	0.6767	-0.23, 0.15	-3.27
Very high risk														.				.				
Risk aversion																						
Very low														.				.				
Low														-0.22	(0.14)	0.1226	-0.50, 0.06	-0.18	(0.10)	0.0572	-0.37, 0.01	-11.21
Moderately														-0.36	(0.16)	0.0206	-0.67, -0.06	-0.15	(0.11)	0.1594	-0.36, 0.06	-10.43
High														-0.53	(0.11)	<.0001	-0.74, -0.32	-0.29	(0.07)	0.0001	-0.44, -0.15	-17.95
Vaccination status																						
No														-4.31	(0.19)	<.0001	-4.68, -3.94	-0.04	(0.27)	0.8767	-0.58, 0.49	-48.96
Not yet, but I intend to														-2.24	(0.25)	<.0001	-2.74, -1.75	-0.30	(0.29)	0.3016	-0.87, 0.27	-32.86
Yes, the first shot														-1.03	(0.21)	<.0001	-1.44, -0.62	-0.18	(0.17)	0.2835	-0.50, 0.15	-16.80
Yes, both shots														-1.02	(0.10)	<.0001	-1.22, -0.82	-0.22	(0.07)	0.0010	-0.36, -0.09	-18.74
Yes, three shots (booster)														.				.				
Peers' vaccination status																						
Most														0.44	(0.26)	0.0905	-0.07 , 0.94	-0.23	(0.25)	0.3541	-0.73, 0.26	-8.85
About half														-0.24	(0.28)	0.3942	-0.78 , 0.31	0.20	(0.27)	0.4494	-0.32, 0.73	9.94
Just a few														0.00	(0.27)	0.9925	-0.54 , 0.53	0.10	(0.26)	0.7008	-0.41, 0.61	5.73
None														.				.				.

AME– Average marginal effect, CI– Confidence Interval, Est.– Estimate, No.– Number, SE– Standard error

In Model 4, which included control variables, the significance and effect sizes mostly decreased for the duration since the most recent HIS and the severity of HIS. Compared to reporting having not experienced any HIS, reporting that the most recent HIS had occurred in the two weeks or in the three to four weeks before the survey increased the WTP for a booster vaccination by €1.92 (logistic: *P* =.0025, gamma: *P* =.8060) and by €8.95 (logistic: *P* =.0095, gamma: *P* =.4427), respectively. These results suggest that having experienced a HIS was associated with being willingness to pay, but not with the height of the WTP (conditional on being positive). If the HIS was reported to have occurred between five and eight weeks before the survey, compared to reporting not having experienced a HIS, the AME decreased (€-4.60 and €1.80, respectively, all *P* >.05). Again, the severity of HIS was negatively associated with being willing to pay if the HIS was reported as having been milder than expected (€-6.14, logistic: 0.0093, gamma: 0.4451) and positively if it was reported as having been a bit more severe (€4.53, logistic: 0.0589, gamma: 0.6595) or more severe than expected (€5.23, logistic: 0.0842, gamma: 0.6625) compared to those who reported that the HIS was as severe or as mild as expected.

## Discussion

### Main findings

When willingness-to-pay (WTP) exercises and other forms of contingent valuation are used to elicit population preferences, the timing of elicitation relative to a relevant event might be associated with the results. To investigate this issue, we analyzed whether recent health information shocks (HIS) were associated with WTP.

The results of our regression analyses suggest that HIS are indeed positively associated with WTP for a booster vaccination for COVID-19, especially during the first four weeks after a HIS. This association may decrease over time but could nonetheless persist over a longer period. Our results also suggest that the association between HIS and WTP was especially pronounced through increasing the *probability* of having a positive WTP (as observed in the logistic regression), more so than with the *height* of the WTP conditional on being positive.

When an individual receives news of a potential exposure in the recent past, it seems plausible that they might place a higher valuation on a preventive measure, such as a booster vaccination. However, we did not measure whether our participants indeed had contact with an infected person. Another explanation could be that participants might re-evaluate the risk of developing COVID-19 themselves and the consequences of contracting it. The fact that the WTP associated with a HIS that occurred from two to four weeks before the survey was higher than the WTP associated with a HIS that occurred from zero to two weeks before the survey might be related to the time it takes for severe symptoms and their consequences to develop.

Experiencing a health information shock, which can be perceived as a threat to one’s own health, might have a lasting consequence on individuals’ risk perceptions and thus their WTP preferences. This is supported by empirical findings from different fields. Evidence from the United States (US), for example, has shown that regional flood insurance purchases are highly correlated with the level of flood damage in the region during the prior year [42]. Similarly, Dave et al. (2020) examined risk perceptions of smoking e-cigarettes before and during an outbreak of e-cigarette or vaping-related lung injuries in the US in 2019 and 2020. They reported that risk perceptions decreased when participants received more information regarding the source of the outbreak, but not to pre-outbreak levels [24]. Other health shocks, like flu outbreaks, have been hypothesized to be associated with sustained changes in hygiene practices in developing countries [43]. Further evidence suggests similar longer-lasting effects [44].

### Limitations

Our study has a number of important limitations related to its setting, sample, measurement, and analysis that must be considered when interpreting its results. First, as we use cross-sectional data here, no causal claim can be made based on our results. Further, latent mechanisms driving both HIS and the booster intention might contribute to the associations observed in this study. For instance, individuals with a higher fear of COVID-19 might have tested themselves and their social environment more often for the virus, thus experiencing more HIS, than individuals with a lower fear of COVID-19. While the variable *risk to own health* attempts to proxy for a fear of COVID-19, other latent variables may have influenced our results, e.g. personality traits.

Second, the Omicron variant of the coronavirus was starting to spread in Europe at the time of data collection. Official European institutions recommended that people should receive a booster shot around the end of 2021 [45–47]. While this highlights the relevance of looking at booster shots in our study, the status of the countries’ national booster campaigns as well as the media coverage differed at the time of data collection. For instance, 48.3 booster shots per 100 people were administered in the UK by the end of 2021 compared to 29.0 booster shots per 100 people in Italy [48]. Additionally, our sample contained a higher proportion of individuals who had received a booster shot than in the general population (except for the participants from Portugal). We accounted for these circumstances by including vaccination status at the individual level and country fixed-effects in Model 4 (see Online Resource for analysis of boosted and not boosted individuals separately). Participants who reported experiencing a HIS may have furthermore incorrectly assumed that they would enjoy immediate protection from a booster shot [49]. This may have resulted in overly high valuations of the booster shot compared to a situation in which people have better information about the real effects of booster vaccinations.

Third, the high share of protest answers in our sample suggests that a substantial number of participants more fundamentally rejected participating in the WTP exercise. In particular, this appeared to be related to respondents expecting or demanding that governments or health insurers should cover the costs of a booster vaccination. Given the abovementioned public efforts in booster campaigns, it might have been difficult for individuals to immerse themselves in the hypothetical setting, which counterfactually states no expected coverage by a third party. Importantly, the majority of protesters appeared to place a positive value on the booster vaccination given that they indicated that another entity should be responsible for covering the costs; this suggests that participants rejected the payment vehicle (i.e., the out-of-pocket payment) rather than the booster vaccination itself. Indeed, observed zero valuations of the good on offer can mean that participants are not willing to pay for the good because they do not associate value with it (i.e., ‘true zeros’), but could also mean that they are not willing to pay because they reject the scenario (i.e., ‘protest zeros’) [50–52]. Protesters are often excluded from samples before analysis [50, 51], which can lead to a loss of sample representativeness or to bias if protesters’ and non-protesters’ characteristics differ significantly [50]. In our study, subsample analyses suggested that the characteristics of protesters were fairly similar to those of non-protesters (i.e., the analyzed sample). We therefore chose not to opt for econometric techniques to correct for this [50]. Indeed, a sensitivity analysis including all protesters in the sample and assigning them a WTP of €0 revealed only minor variations in estimates and significance compared to Model 4 due to the two-part model’s separate handling of zeros and non-negative values (see Online Resource).

Fourth, in our dataset we could not distinguish between experiencing a health shock (i.e., a COVID-19 infection) oneself or within one’s household, thus, between two quite distinct situations. Also, it is important to emphasize that we chose not to incorporate the degree of personal connection as a variable in our final models because the model selection criteria suggested that there was higher model complexity when including the variable but only minimal information gain.

Finally, we chose to examine the most recent HIS in the analysis because we assumed that this shock was the most relevant for the valuation of the booster shot as a preventive countermeasure. Furthermore, we controlled for the number of HIS as a covariate. Some individuals expressed having experienced multiple HIS in our survey. In these cases, we did not measure the severity of each HIS separately but rather asked the participants to state the severity of the majority of HIS they experienced among people they know (including themselves). When we excluded individuals who experienced multiple HIS, the peak in the increase in WTP for those experiencing the HIS three to four weeks before the elicitation exercise was approximately €5 lower, and the association of the severity of HIS with the WTP was lower.

### Implications

Although WTP is a well-established instrument in health economics, methodological challenges remain. Our results suggest that recent health information that is relevant to the goods being valued can be associated with having a positive WTP in certain cases in the short term, especially by serving as a ‘nudge’ to decide if these goods are worth paying for. Researchers and policymakers may therefore wish to consider the timing of valuation exercises as well as how this relates to potential HIS. Finding the optimal point in time to measure WTP is difficult and may depend on the policy question under consideration, so that *the* optimal point in general may not exist. Given our findings, researchers may wish to elicit information about HIS in situations where they are likely to occur, aiding the interpretation of their findings.

From a population perspective, the probability of relevant HIS occurring may differ depending on the disease being studied. For example, the prevalence of diabetes mellitus among adults in Europe is high [53], so the probability of experiencing a HIS due to someone or oneself being diagnosed with diabetes or experiencing consequences of the disease may be comparatively high as well [54]. In contrast, the probability of experiencing a HIS related to rare conditions may, by definition, be considerably lower. If WTP values differ because the frequency of HIS varies depending on the disease, policy makers may wish to consider potential distributional consequences in their subsequent decision making.

## Conclusion

We found that timing may matter when measuring WTP because relevant events, like health information shocks (HIS), may be associated with WTP. In our study, HIS appeared especially related to the probability of having a positive WTP (i.e., more people willing to pay for a booster) and less with the height of the WTP conditional on being willing to pay. Moreover, the association of HIS with WTP was especially visible in the short term but may also persist over the long term. Our findings suggest that researchers and policymakers should be aware of potential HIS when eliciting and interpreting results obtained through WTP exercises and other forms of contingent valuation.

## Electronic supplementary material

Below is the link to the electronic supplementary material.

Supplementary Material 1

## References

[1] Diener, A., O’Brien, B., Gafni, A.: Health care contingent valuation studies: A review and classification of the literature. Health Econ. **7**, 313–326 (1998) 9683092 10.1002/(sici)1099-1050(199806)7:4<313::aid-hec350>3.0.co;2-b

[2] Bala, M.V., Mauskopf, J.A., Wood, L.L.: Willingness to pay as a measure of health benefits. PharmacoEconomics. **15**, 9–18 (1999) 10345161 10.2165/00019053-199915010-00002

[3] Klose, T.: The contingent valuation method in health care. Health Policy. **47**, 97–123 (1999) 10538292 10.1016/s0168-8510(99)00010-x

[4] Bayoumi, A.M.: The measurement of contingent valuation for health economics. PharmacoEconomics. **22**, 691–700 (2004) 15250748 10.2165/00019053-200422110-00001

[5] Olsen, J.A., Smith, R.D.: Theory versus practice: A review of ‘willingness-to-pay’ in health and health care. Health Econ. **10**, 39–52 (2001) 11180568 10.1002/1099-1050(200101)10:1<39::aid-hec563>3.0.co;2-e

[6] Lin, P.-J., Cangelosi, M.J., Lee, D.W., Neumann, P.J.: Willingness to pay for diagnostic technologies: A review of the contingent valuation literature. Value Health. **16**, 797–805 (2013) 23947973 10.1016/j.jval.2013.04.005

[7] Wolff, E., Larsson, S., Svensson, M.: Willingness to pay for health improvements using stated preferences: Prevention versus treatment. Value Health. **23**, 1384–1390 (2020) 33032783 10.1016/j.jval.2020.06.008

[8] Bobinac, A., van Exel, N.J.A., Rutten, F.F., Brouwer, W.B.: GET MORE, PAY MORE? An elaborate test of construct validity of willingness to pay per QALY estimates obtained through contingent valuation. J. Health Econ. **31**, 158–168 (2012) 22018622 10.1016/j.jhealeco.2011.09.004

[9] Kanya, L., Sanghera, S., Lewin, A., Fox-Rushby, J.: The criterion validity of willingness to pay methods: A systematic review and meta-analysis of the evidence. Soc. Sci. Med. **232**, 238–261 (2019) 31108330 10.1016/j.socscimed.2019.04.015

[10] Donaldson, C., Thomas, R., Torgerson, D.J.: Validity of open-ended and payment scale approaches to eliciting willingness to pay. Appl. Econ. **29**, 79–84 (1997)

[11] Frew, E.J., Whynes, D.K., Wolstenholme, J.L.: Eliciting willingness to pay: Comparing Closed-Ended with Open-Ended and payment scale formats. Med. Decis. Mak. **23**, 150–159 (2003) 10.1177/0272989X03251245 12693877

[12] Frew, E.J., Wolstenholme, J.L., Whynes, D.K.: Comparing willingness-to-pay: Bidding game format versus open-ended and payment scale formats. Health Policy. **68**, 289–298 (2004) 15113640 10.1016/j.healthpol.2003.10.003

[13] Ryan, M., Scott, D.A., Donaldson, C.: Valuing health care using willingness to pay: A comparison of the payment card and dichotomous choice methods. J. Health Econ. **23**, 237–258 (2004) 15019754 10.1016/j.jhealeco.2003.09.003

[14] Whynes, D.K., Wolstenholme, J.L., Frew, E.: Evidence of range bias in contingent valuation payment scales. Health Econ. **13**, 183–190 (2004) 14737755 10.1002/hec.809

[15] Shackley, P., Dixon, S.: The random card sort method and respondent certainty in contingent valuation: An exploratory investigation of range bias. Health Econ. **23**, 1213–1223 (2014) 23922327 10.1002/hec.2980

[16] Gyrd-Hansen, D., Jensen, M.L., Kjaer, T.: Framing the willingness-to-pay question: Impact on response patterns and mean willingness to pay. Health Econ. **23**, 550–563 (2014) 23696155 10.1002/hec.2932

[17] Ahlert, M., Breyer, F., Schwettmann, L.: How you ask is what you get: Framing effects in willingness-to-pay for a QALY. Soc. Sci. Med. **150**, 40–48 (2016) 26730880 10.1016/j.socscimed.2015.11.055

[18] Soeteman, L., van Exel, J., Bobinac, A.: The impact of the design of payment scales on the willingness to pay for health gains. Eur. J. Health Econ. **18**, 743–760 (2017) 27623946 10.1007/s10198-016-0825-y PMC5486460

[19] Versteegh, M.M., Brouwer, W.: Patient and general public preferences for health States: A call to reconsider current guidelines. Soc. Sci. Med. **165**, 66–74 (2016) 27497260 10.1016/j.socscimed.2016.07.043

[20] Peeters, Y., Stiggelbout, A.M.: Health state valuations of patients and the general public analytically compared: A Meta-Analytical comparison of patient and population health state utilities. Value Health. **13**, 306–309 (2010) 19744288 10.1111/j.1524-4733.2009.00610.x

[21] Dolan, P., Kahneman, D.: Interpretations of utility and their implications for the valuation of health. Econ. J. **118**, 215–234 (2008)

[22] Menzel, P., Dolan, P., Richardson, J., Olsen, J.A.: The role of adaptation to disability and disease in health state valuation: A preliminary normative analysis. Soc. Sci. Med. **55**, 2149–2158 (2002) 12409128 10.1016/s0277-9536(01)00358-6

[23] Jensen, V.M., Wüst, M.: Can caesarean section improve child and maternal health? The case of breech babies. J. Health Econ. **39**, 289–302 (2015) 25179865 10.1016/j.jhealeco.2014.07.004

[24] Dave, D., Dench, D., Kenkel, D., Mathios, A., Wang, H.: News that takes your breath away: Risk perceptions during an outbreak of vaping-related lung injuries. J. Risk Uncertain. **60**, 281–307 (2020) 34504389 10.1007/s11166-020-09329-2 PMC8425473

[25] Larsen, V.B., Grøsland, M., Telle, K., Magnusson, K.: Utilization of health care services before and after media attention about fatal side effects of the AstraZeneca vaccine: A nation-wide register-based event study. BMC Health Serv. Res. **21**, 1229 (2021) 34774045 10.1186/s12913-021-07233-2 PMC8590367

[26] Wu, B., David, G.: Information, relative skill, and technology abandonment. J. Health Econ. **83**, 102596 (2022) 35303551 10.1016/j.jhealeco.2022.102596

[27] Islam, M.N., Rabbani, A., Sarker, M.: Health shock and preference instability: Assessing health-state dependency of willingness-to-pay for corrective eyeglasses. Health Econ. Rev. **9**, 32 (2019) 31696342 10.1186/s13561-019-0249-3 PMC6836482

[28] Our World in Data: Cumulative confirmed COVID-19 cases. Data source: World Health Organization COVID-19 Dashboard: (2023). https://ourworldindata.org/explorers/coronavirus-data-explorer?zoomToSelection=true%26;time=2021-12-22.2022-01-11%26;facet=none%26;pickerSort=desc%26;pickerMetric=new_cases_smoothed_per_million%26;Metric=Confirmed+cases%26;Interval=Cumulative%26;Relative+to+Population=true%26;Color+by+test+positivity=false%26;country=GBR~DEU~FRA~ITA~NLD~ESP~DNK. Accessed 23 Aug 2023

[29] Our World in Data: SARS-CoV-2 variants in analyzed sequences: Data source: GISAID, via CoVariants.org: (2024). https://ourworldindata.org/grapher/covid-variants-area?time=2021-12-20.2022-01-17%26;country=GBR~DNK~FRA~DEU~ITA~NLD~ESP. Accessed 17 Apr 2024

[30] Sabat, I., Neumann-Böhme, S., Varghese, N.E., Barros, P.P., Brouwer, W., van Exel, J., et al.: United but divided: Policy responses and People’s perceptions in the EU during the COVID-19 outbreak. Health Policy. **124**, 909–918 (2020) 32631613 10.1016/j.healthpol.2020.06.009 PMC7307992

[31] Varghese, N.E., Sabat, I., Neumann-Böhme, S., Schreyögg, J., Stargardt, T., Torbica, A., et al.: Risk communication during COVID-19: A descriptive study on familiarity with, adherence to and trust in the WHO preventive measures. PLOS ONE. **16**, e0250872 (2021) 33914814 10.1371/journal.pone.0250872 PMC8084201

[32] Hajek, A., Sabat, I., Neumann-Böhme, S., Schreyögg, J., Barros, P.P., Stargardt, T., König, H.-H.: Prevalence and determinants of probable depression and anxiety during the COVID-19 pandemic in seven countries: Longitudinal evidence from the European COvid survey (ECOS). J. Affect. Disord. **299**, 517–524 (2022) 34920039 10.1016/j.jad.2021.12.029 PMC8684990

[33] Sabat, I., Neumann-Böhme, S., Pita Barros, P., Brinkmann, C., Brouwer, W., van Exel, J., et al.: The European COvid Survey (ECOS): Technical Report (2024). https://www.hche.uni-hamburg.de/dokumente/research-papers/rp30-ecos-technical-paper.pdf. Accessed 17 Apr 2024

[34] Himmler, S., van Exel, J., Perry-Duxbury, M., Brouwer, W.: Willingness to pay for an early warning system for infectious diseases. Eur. J. Health Econ. **21**, 763–773 (2020) 32180067 10.1007/s10198-020-01171-2 PMC7364296

[35] Eurostat Database: Adjusted gross disposable income of households per capita (Code: sdg_10_20). Accessed 9 Dec 2022.

[36] Eurostat Database: Purchasing power adjusted GDP per capita (Code: sdg_10_10). Accessed 22 Jul 2022

[37] Zhang, C., Conrad, F.: Speeding in web surveys: The tendency to answer very fast and its association with straightlining. Surv. Res. Methods **8** (2014)

[38] Donaldson, C., Jones, A.M., Mapp, T.J., Olson, J.A.: Limited dependent variables in willingness to pay studies: Applications in health care. Appl. Econ. **30**, 667–677 (1998)

[39] Steigenberger, C., Flatscher-Thoeni, M., Siebert, U., Leiter, A.M.: Determinants of willingness to pay for health services: A systematic review of contingent valuation studies. Eur. J. Health Econ. **23**, 1455–1482 (2022) 35166973 10.1007/s10198-022-01437-x PMC8853086

[40] Barsky, R.B., Juster, F.T., Kimball, M.S., Shapiro, M.D.: Preference parameters and behavioral heterogeneity: An experimental approach in the health and retirement study. Q. J. Econ. **112**, 537–579 (1997)

[41] Belotti, F., Deb, P., Manning, W.G., Norton, E.C.: Twopm: Two-Part models. Stata J. **15**, 3–20 (2015)

[42] Browne, M.J., Hoyt, R.E.: The demand for flood insurance: Empirical evidence. J. Risk Uncertain. **20**, 291–306 (2000)

[43] Agüero, J.M., Beleche, T.: Health shocks and their long-lasting impact on health behaviors: Evidence from the 2009 H1N1 pandemic in Mexico. J. Health Econ. **54**, 40–55 (2017) 28414953 10.1016/j.jhealeco.2017.03.008 PMC7114327

[44] Sundmacher, L.: The effect of health shocks on smoking and obesity. Eur. J. Health Econ. **13**, 451–460 (2012) 21559942 10.1007/s10198-011-0316-0

[45] European Centre for Disease Prevention and Control: Assessment of the further emergence and potential impact of the SARS-CoV-2 Omicron variant of concern in the context of ongoing transmission of the Delta variant of concern in the EU/EEA, 18th update– 15 December 2021. Stockholm: (2021)

[46] European Centre for Disease Prevention and Control: Communicable disease threats report, 2–8 January 2022, week 1–07 January 2022. Stockholm: (2022)

[47] European Centre for Disease Prevention and Control: Communicable disease threats report, 9–15 January 2022, week 2–14 January 2022. Stockholm: (2022)

[48] Our World in Data: COVID-19 vaccine boosters administered per 100 people: (2022). https://ourworldindata.org/grapher/covid-vaccine-booster-doses-per-capita?time=2021-12-24.2022-01-11%26;country=DEU~GBR~ESP~PRT~NLD~ITA~DNK~FRA. Accessed 25 Oct 2022

[49] Barda, N., Dagan, N., Cohen, C., Hernán, M.A., Lipsitch, M., Kohane, I.S., et al.: Effectiveness of a third dose of the BNT162b2 mRNA COVID-19 vaccine for preventing severe outcomes in Israel: An observational study. Lancet. **398**, 2093–2100 (2021) 34756184 10.1016/S0140-6736(21)02249-2 PMC8555967

[50] Rankin, J., Robinson, A.: Accounting for Protest zeros in Contingent Valuation Studies: A Review of Literature. University of East Anglia, Health Economics Group (HEG), Norwich (2018)

[51] Frey, U.J., Pirscher, F.: Distinguishing protest responses in contingent valuation: A conceptualization of motivations and attitudes behind them. PLOS ONE. **14**, e0209872 (2019) 30620731 10.1371/journal.pone.0209872 PMC6324805

[52] Sendi, P., Ramadani, A., Bornstein, M.M.: Prevalence of missing values and protest zeros in contingent valuation in dental medicine. Int. J. Environ. Res. Public. Health **18** (2021) 10.3390/ijerph18147219 PMC8307611 34299670

[53] International Diabetes Foundation: IDF Diabetes Atlas: Europe: (2019). https://www.diabetesatlas.org/data/upload/download/eur_factsheet_en.pdf. Accessed 28 Oct 2022

[54] Einarson, T.R., Acs, A., Ludwig, C., Panton, U.H.: Prevalence of cardiovascular disease in type 2 diabetes: A systematic literature review of scientific evidence from across the world in 2007–2017. Cardiovasc. Diabetol. **17**, 83 (2018) 29884191 10.1186/s12933-018-0728-6 PMC5994068

