# Supplementary material for: Does timing matter? The role of health information shocks in measuring willingness to pay
Source: Eur J Health Econ. 2025 Apr 17;26(8):1401–13. doi: 10.1007/s10198-025-01774-7 (PMC12572092; doi:10.1007/s10198-025-01774-7)
Supplement: Supplementary file 1 — Supplementary Material 1 [file 10198_2025_1774_MOESM1_ESM.docx]

**Does Timing Matter? The Role of Health Information Shocks in Measuring Willingness to Pay**

The European Journal of Health Economics

Brinkmann, C^1^; Neumann-Böhme, S^1,2^; Brouwer, WBF^2^; Stargardt, T^1^.

^1^ Hamburg Center for Health Economics, University of Hamburg, Hamburg, Germany

^2^ Erasmus School of Health Policy and Management, Erasmus University Rotterdam, Rotterdam, the Netherlands

Appendix 1: Matrix used to measure HIS


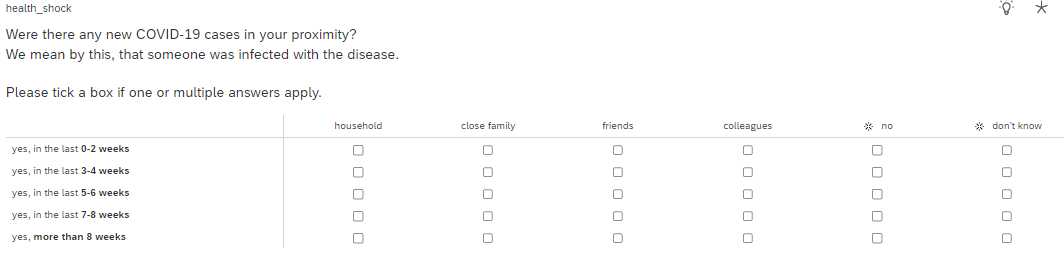


Appendix 2: WTP elicitation procedure


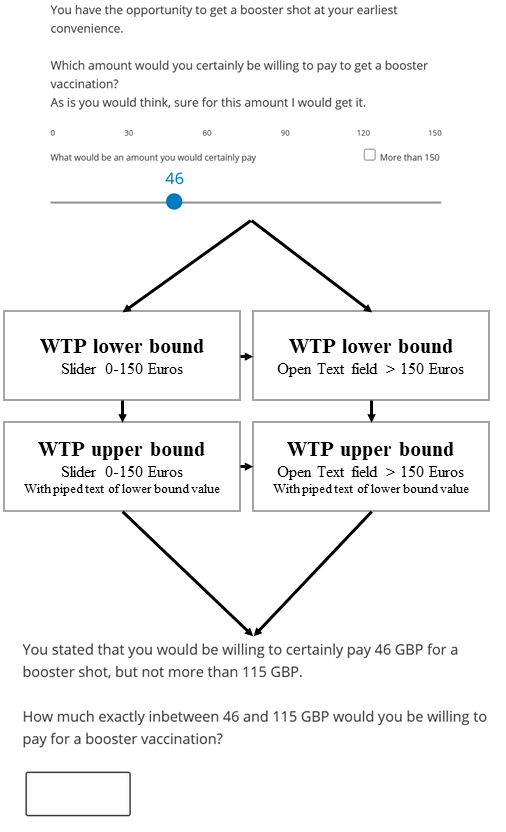


Appendix 3: Educational qualification and assigned education level

| **Country** | **Education level** | **Educational qualification/ educational institution** |
| --- | --- | --- |
| Denmark | Low | Folkeskolen - f.eks. 9. eller 10. klasse |
|  | Middle | Gymnasial uddannelse - f.eks. Almen Gymnasium, HHX, HTX osv. |
|  | High | En videregående erhvervsuddannelse - f.eks. landbrugs-, social- og sundheds uddannelser, produktionsskole  En mellemlang videregående uddannelse  Universitets uddannelse |
| France | Low | École Primaire  Collège |
|  | Middle | Lycée d’Enseignement général et technologique  Lycée professionnel |
|  | High | Grande École de Commerce et de Gestion/ scientifique, Établissement d`Enseignement supérieur catholique/ artistique/ agricole  Grand Établissement/ École normale supérieure/ d`Ingénieur/ d`Architecture/ nationale vétérinaire  Université, Institut universitaire de Technologie/ national polytechnique/ d’Études politiques/ universitaire de Formation des Maîtres |
| Germany | Low | Grundschule  Hauptschule  Realschule |
|  | Middle | Gymnasium/ Berufliches Gymnasium/ Fachgymnasium, Gesamtschule  Fachoberschule, Fachschule, Berufsschule, Berufsfachschule |
|  | High | Technische Hochschule, Pädagogische Hochschule, Kunsthochschule/ Musikhochschule  Fachhochschule  Universität, Technische Universität |
| Italy | Low | Scuola elementare  Scuola media inferiore |
|  | Middle | Istituto professionale  Scuola superiore |
|  | High | Università  Master  Dottorato |
| Netherlands | Low | LO (lagere school, LAVO, VGLO)  LBO (LBO, LTS, ITO, LEAO, Huishoudschool, LLO)  MAO (MAVO, IVO, MULO, ULO, 3jr HBS, 3jr VWO, 3jr VHMO) |
|  | Middle | MBO (MTS, UTS, MEAO)  HAO (HAVO, VWO, Atheneum, Gymnasium, NMS, HBS, Lyceum) |
|  | High | HBO (HTS, HEAO, Wetensch. kand., Univers. onderwijs kand.)  WO (Universitair onderwijs, Doctoraalopleiding, TH) |
| Portugal | Low | Sem Estudos  Primário Incompleto  Primário Completo |
|  | Middle | Nível Médio Incompleto  Nível Médio Completo |
|  | High | Superior Incompleto  Superior Completo |
| Spain | Low | Educación Primaria / EGB  Educación Secundaria Obligatoria (ESO) / BUP  Formación Profesional Básica |
|  | Middle | Bachillerato / Curso de Orientación Universitaria (COU)  Formación Profesional de Grado Medio |
|  | High | Ciclo formativo de Grado Superior  Licenciatura / Diplomatura / Ingeniería/ Grado / Máster  Tercer Ciclo / Doctorado |
| United Kingdom | Low | Combined Junior and Infant School/ Infant School  Junior School  Comprehensive School |
|  | Middle | Comprehensive School (GCSE)/ Secondary Modern (GCSE)/ Grammar School (GCSE)/ City Technology College (CGSE)/ Sixth Form |
|  | High | College and Institution of Higher education  Open College -College of Technology - Institute/ Teacher Training College  University/ Open University |

**Denmark**: F.eks. - for eksempel, HHX - Hojere Handelseksamen, HTX - Højere Teknisk Eksamen,

**Netherlands**: HAO - Hoger Algemeen Onderwijs, HAVO - Hoger Algemeen Voortgezet Onderwijs, HBO - Hoger beroepsonderwijs, HBS - Hogere Burgerschool, HEAO - Hoger Economisch en Administratief Onderwijs, HTS - Hogere Technische School, ITO - Individueel Technisch Onderwijs, IVO - Individueel Voortgezet Onderwijs, Kand. – Kandidaat, LAVO - Lagere Algemeen Voortgezet Onderwijs, LBO - Lager beroepsonderwijs, LEAO - Lager Economisch en Administratief Onderwijs, LLO - Leven Lang Ontwikkelen, LO - Lager Onderwijs, LTS - Lagere Technische School, MAO - Middelbaar Algemeen Onderwijs, MAVO - Middelbaar Algemeen Voortgezet Onderwijs, MBO - Middelbaar beroepsonderwijs, MEAO - Middelbaar Economisch en Administratief Onderwijs, MULO - Meer Uitgebreid Lager Onderwijs, MTS - Middelbare Technische School, NMS - Nederlandse Middelbare School, TH - technische hogeronderwijsinstellingen, ULO - Uitgebreid Lager Onderwijs, UTS - Uitgebreide Technische School, VGLO - Voortgezet Gewoon Lager Onderwijs, VHMO - Voorbereidend Hoger en Middelbaar Onderwijs, VWO - Voorbereidend Wetenschappelijk Onderwijs, Wetensch. – wetenschappelijk, WO - Wetenschappelijk Onderwijs,

**Spain**: BUP - Bachillerato Unificado Polivalente, EGB - Educación General Básica,

**United** **Kingdom**: CGSE - College and Graduate School of Education, GCSE - General Certificate of Secondary Education

Appendix 4: Sensitivity analysis including protesters

|  | Sensitivity analysis - Full sample including protesters | | | | | | | | | | | | |
| --- | --- | --- | --- | --- | --- | --- | --- | --- | --- | --- | --- | --- | --- |
|  | Logistic | | | | | | Gamma | | | | | | AME |
|  | Est. | SE | p value | 95%CI | | | Est. | SE | p value | 95%CI | | | Est. |
| Intercept | 0.35 | (0.26) | 0.1820 | -0.16 | - | 0.86 | 4.44 | (0.31) | <.0001 | 3.83 | - | 5.05 |  |
| No. Of HIS | 0.05 | (0.02) | 0.0007 | 0.02 | - | 0.08 | 0.03 | (0.02) | 0.0401 | 0.00 | - | 0.07 | 1.74 |
| Temporal proximity |  |  |  |  |  |  |  |  |  |  |  |  |  |
| 0-2 weeks since HIS | 0.38 | (0.10) | 0.0001 | 0.20 | - | 0.57 | -0.03 | (0.10) | 0.8060 | -0.23 | - | 0.18 | 3.69 |
| 3-4 weeks since HIS | 0.29 | (0.12) | 0.0158 | 0.05 | - | 0.52 | 0.10 | (0.13) | 0.4427 | -0.16 | - | 0.36 | 7.21 |
| 5-6 weeks since HIS | 0.31 | (0.15) | 0.0364 | 0.02 | - | 0.59 | -0.16 | (0.16) | 0.3311 | -0.47 | - | 0.16 | -1.39 |
| 7-8 weeks since HIS | 0.36 | (0.20) | 0.0746 | -0.04 | - | 0.76 | 0.00 | (0.22) | 0.9976 | -0.42 | - | 0.43 | 4.37 |
| More than 8 weeks since HIS | 0.25 | (0.12) | 0.0397 | 0.01 | - | 0.48 | 0.02 | (0.13) | 0.8997 | -0.25 | - | 0.28 | 3.58 |
| No HIS | . |  |  |  |  |  | . |  |  |  |  |  | . |
| Severity of HIS |  |  |  |  |  |  |  |  |  |  |  |  |  |
| Milder than expected | -0.35 | (0.09) | 0.0001 | -0.53 | - | -0.18 | -0.08 | (0.10) | 0.4451 | -0.27 | - | 0.12 | -6.49 |
| A bit milder than expected | 0.07 | (0.10) | 0.4620 | -0.12 | - | 0.26 | 0.01 | (0.10) | 0.8871 | -0.18 | - | 0.21 | 1.31 |
| As severe/mild as expected |  |  |  |  |  |  |  |  |  |  |  |  |  |
| A bit more severe than expected | 0.37 | (0.11) | 0.0008 | 0.15 | - | 0.58 | 0.05 | (0.11) | 0.6595 | -0.16 | - | 0.26 | 6.01 |
| More severe than expected | 0.41 | (0.13) | 0.0023 | 0.15 | - | 0.67 | 0.06 | (0.13) | 0.6625 | -0.20 | - | 0.32 | 6.88 |
| Mixed, i.e. half more severe, half milder than expected | -0.16 | (0.13) | 0.2252 | -0.43 | - | 0.10 | -0.06 | (0.15) | 0.6679 | -0.35 | - | 0.23 | -3.90 |
| No HIS | . |  |  |  |  |  | . |  |  |  |  |  | . |
| Female | -0.01 | (0.05) | 0.8067 | -0.11 | - | 0.09 | -0.02 | (0.06) | 0.7070 | -0.13 | - | 0.09 | -0.86 |
| Age |  |  |  |  |  |  |  |  |  |  |  |  |  |
| 18-24 years old | -0.42 | (0.12) | 0.0002 | -0.65 | - | -0.20 | 0.22 | (0.14) | 0.1067 | -0.05 | - | 0.48 | 2.98 |
| 25-34 years old | -0.41 | (0.09) | <.0001 | -0.60 | - | -0.23 | 0.02 | (0.11) | 0.8248 | -0.18 | - | 0.23 | -3.90 |
| 35-44 years old | -0.43 | (0.09) | <.0001 | -0.60 | - | -0.26 | 0.02 | (0.10) | 0.8023 | -0.16 | - | 0.21 | -4.10 |
| 45-54 years old | -0.50 | (0.08) | <.0001 | -0.66 | - | -0.34 | -0.01 | (0.09) | 0.9420 | -0.18 | - | 0.17 | -5.93 |
| 55-64 years old | -0.19 | (0.08) | 0.0234 | -0.35 | - | -0.03 | -0.04 | (0.09) | 0.6790 | -0.21 | - | 0.13 | -3.28 |
| 65 years old and older | . |  |  |  |  |  | . |  |  |  |  |  | . |
| Education level |  |  |  |  |  |  |  |  |  |  |  |  |  |
| High | 0.13 | (0.08) | 0.0972 | -0.02 | - | 0.29 | 0.15 | (0.09) | 0.0940 | -0.03 | - | 0.33 | 6.33 |
| Middle | -0.05 | (0.08) | 0.5401 | -0.20 | - | 0.11 | 0.19 | (0.09) | 0.0423 | 0.01 | - | 0.37 | 5.41 |
| Low | . |  |  |  |  |  | . |  |  |  |  |  | . |
| Level of making ends meet |  |  |  |  |  |  |  |  |  |  |  |  |  |
| Easily | 1.28 | (0.12) | <.0001 | 1.04 | - | 1.51 | 0.47 | (0.14) | 0.0011 | 0.19 | - | 0.75 | 32.87 |
| Fairly easy | 0.86 | (0.10) | <.0001 | 0.66 | - | 1.06 | 0.16 | (0.13) | 0.2140 | -0.09 | - | 0.42 | 14.09 |
| With some difficulty | 0.35 | (0.10) | 0.0005 | 0.15 | - | 0.55 | 0.08 | (0.13) | 0.5511 | -0.18 | - | 0.33 | 5.67 |
| With great difficulty | . |  |  |  |  |  | . |  |  |  |  |  | . |
| Country |  |  |  |  |  |  |  |  |  |  |  |  |  |
| Denmark | 0.20 | (0.11) | 0.0577 | -0.01 | - | 0.41 | -0.04 | (0.11) | 0.7088 | -0.27 | - | 0.18 | 0.82 |
| Spain | -0.19 | (0.10) | 0.0675 | -0.39 | - | 0.01 | -0.43 | (0.12) | 0.0002 | -0.66 | - | -0.20 | -14.09 |
| France | 0.09 | (0.10) | 0.3755 | -0.11 | - | 0.28 | -0.47 | (0.11) | <.0001 | -0.69 | - | -0.24 | -12.75 |
| Italy | 0.03 | (0.10) | 0.7776 | -0.17 | - | 0.23 | -0.36 | (0.11) | 0.0018 | -0.58 | - | -0.13 | -10.44 |
| Netherlands | -0.29 | (0.10) | 0.0049 | -0.48 | - | -0.09 | -0.02 | (0.12) | 0.8371 | -0.26 | - | 0.21 | -4.16 |
| Portugal | 0.42 | (0.11) | 0.0001 | 0.22 | - | 0.63 | -0.80 | (0.12) | <.0001 | -1.02 | - | -0.57 | -17.59 |
| United Kingdom | 0.04 | (0.10) | 0.6894 | -0.16 | - | 0.24 | -0.16 | (0.11) | 0.1528 | -0.38 | - | 0.06 | -4.70 |
| Germany | . |  |  |  |  |  | . |  |  |  |  |  | . |
| Health Problematization Index | 0.03 | (0.01) | 0.0008 | 0.01 | - | 0.05 | 0.01 | (0.01) | 0.3697 | -0.01 | - | 0.03 | 0.63 |
| Risk to own health from COVID-19 |  |  |  |  |  |  |  |  |  |  |  |  |  |
| No risk at all | -0.90 | (0.12) | <.0001 | -1.14 | - | -0.65 | -0.31 | (0.15) | 0.0393 | -0.60 | - | -0.01 | -19.44 |
| Little risk | -0.47 | (0.10) | <.0001 | -0.66 | - | -0.28 | -0.05 | (0.11) | 0.6249 | -0.26 | - | 0.15 | -7.82 |
| Moderate risk | -0.22 | (0.09) | 0.0155 | -0.40 | - | -0.04 | -0.14 | (0.09) | 0.1313 | -0.33 | - | 0.04 | -7.80 |
| High risk | -0.04 | (0.10) | 0.6900 | -0.23 | - | 0.15 | -0.04 | (0.10) | 0.6767 | -0.23 | - | 0.15 | -2.10 |
| Very high risk | . |  |  |  |  |  | . |  |  |  |  |  | . |
| Risk aversion |  |  |  |  |  |  |  |  |  |  |  |  |  |
| Very low | . |  |  |  |  |  | . |  |  |  |  |  | . |
| Low | -0.38 | (0.09) | <.0001 | -0.54 | - | -0.21 | -0.18 | (0.10) | 0.0572 | -0.37 | - | 0.01 | -11.83 |
| Moderately | -0.31 | (0.10) | 0.0010 | -0.50 | - | -0.13 | -0.15 | (0.11) | 0.1594 | -0.36 | - | 0.06 | -9.91 |
| High | -0.56 | (0.07) | <.0001 | -0.69 | - | -0.44 | -0.29 | (0.07) | 0.0001 | -0.44 | - | -0.15 | -17.28 |
| Vaccination status |  |  |  |  |  |  |  |  |  |  |  |  |  |
| No | -3.09 | (0.16) | <.0001 | -3.41 | - | -2.76 | -0.04 | (0.27) | 0.8767 | -0.58 | - | 0.49 | -37.58 |
| Not yet, but I intend to | -1.74 | (0.20) | <.0001 | -2.13 | - | -1.35 | -0.30 | (0.29) | 0.3016 | -0.87 | - | 0.27 | -29.92 |
| Yes, the first shot | -0.75 | (0.14) | <.0001 | -1.02 | - | -0.48 | -0.18 | (0.17) | 0.2835 | -0.50 | - | 0.15 | -16.31 |
| Yes, both shots | -0.60 | (0.06) | <.0001 | -0.71 | - | -0.48 | -0.22 | (0.07) | 0.0010 | -0.36 | - | -0.09 | -15.73 |
| Yes, three shots (booster) | . |  |  |  |  |  | . |  |  |  |  |  | . |
| Peers' vaccination status |  |  |  |  |  |  |  |  |  |  |  |  |  |
| Most | -0.01 | (0.20) | 0.9781 | -0.40 | - | 0.39 | -0.23 | (0.25) | 0.3541 | -0.73 | - | 0.26 | -8.39 |
| About half | -0.29 | (0.22) | 0.1724 | -0.72 | - | 0.13 | 0.20 | (0.27) | 0.4494 | -0.32 | - | 0.73 | 4.05 |
| Just a few | 0.19 | (0.21) | 0.3845 | -0.23 | - | 0.60 | 0.10 | (0.26) | 0.7008 | -0.41 | - | 0.61 | 6.96 |
| None | . |  |  |  |  |  | . |  |  |  |  |  | . |

AME - Average marginal effect, Est. - Estimate, No. - Number, SE - Standard error

*Appendix 5: Sensitivity analysis analyzing the boosted and not boosted individuals separately*

|  | Sensitivity analysis - Only boosted individuals (n=2847) | | | | | | | | | | | | | Sensitivity analysis - Only not boosted individuals (n=2962) | | | | | | | | | | | | |
| --- | --- | --- | --- | --- | --- | --- | --- | --- | --- | --- | --- | --- | --- | --- | --- | --- | --- | --- | --- | --- | --- | --- | --- | --- | --- | --- |
|  | Logistic | | | | | | Gamma | | | | | | AME | Logistic | | | | | | Gamma | | | | | | AME |
|  | Est. | SE | p value | 95% CI | | | Est. | SE | p value | 95% CI | | | Est. | Est. | SE | p value | 95% CI | | | Est. | SE | p value | 95% CI | | | Est. |
| Intercept | -0.07 | (0.71) | 0.9228 | -1.47 | - | 1.33 | 4.11 | (0.63) | <.0001 | 2.88 |  | 5.35 |  | -2.39 | (0.46) | <.0001 | -3.29 | - | -1.49 | 4.67 | (0.23) | <.0001 | 4.21 | - | 5.12 |  |
| No. Of HIS | 0.16 | (0.06) | 0.0060 | 0.05 | - | 0.27 | 0.03 | (0.02) | 0.1474 | -0.01 | - | 0.08 | 2.86 | 0.10 | (0.03) | 0.0025 | 0.04 | - | 0.17 | 0.03 | (0.01) | 0.0262 | 0.00 | - | 0.05 | 1.58 |
| Temporal proximity |  |  |  |  |  |  |  |  |  |  |  |  |  |  |  |  |  |  |  |  |  |  |  |  |  |  |
| 0-2 weeks since HIS | 0.22 | (0.29) | 0.4575 | -0.35 | - | 0.79 | -0.03 | (0.14) | 0.7985 | -0.30 | - | 0.23 | -1.13 | 0.59 | (0.20) | 0.0038 | 0.19 | - | 0.98 | 0.04 | (0.08) | 0.5950 | -0.12 | - | 0.20 | 5.74 |
| 3-4 weeks since HIS | 0.14 | (0.36) | 0.6943 | -0.56 | - | 0.84 | 0.17 | (0.17) | 0.3217 | -0.17 | - | 0.52 | 12.56 | 0.77 | (0.27) | 0.0043 | 0.24 | - | 1.30 | 0.05 | (0.10) | 0.6585 | -0.16 | - | 0.25 | 7.04 |
| 5-6 weeks since HIS | -0.22 | (0.41) | 0.5847 | -1.03 | - | 0.58 | -0.15 | (0.21) | 0.4706 | -0.56 | - | 0.26 | -9.67 | 0.70 | (0.32) | 0.0283 | 0.07 | - | 1.32 | -0.08 | (0.13) | 0.5458 | -0.33 | - | 0.17 | 2.16 |
| 7-8 weeks since HIS | 0.91 | (0.81) | 0.2569 | -0.67 | - | 2.50 | -0.10 | (0.29) | 0.7354 | -0.66 | - | 0.47 | -2.65 | 0.11 | (0.39) | 0.7838 | -0.67 | - | 0.88 | 0.16 | (0.16) | 0.3441 | -0.17 | - | 0.48 | 6.31 |
| More than 8 weeks since HIS | 0.43 | (0.38) | 0.2531 | -0.31 | - | 1.18 | 0.13 | (0.17) | 0.4622 | -0.21 | - | 0.47 | 10.57 | 0.11 | (0.25) | 0.6668 | -0.38 | - | 0.60 | -0.08 | (0.11) | 0.4508 | -0.30 | - | 0.13 | -1.75 |
| No HIS | . |  |  |  |  |  | . |  |  |  |  |  | . | . |  |  |  |  |  | . |  |  |  |  |  | . |
| Severity of HIS |  |  |  |  |  |  |  |  |  |  |  |  |  |  |  |  |  |  |  |  |  |  |  |  |  |  |
| Milder than expected | -0.07 | (0.30) | 0.8080 | -0.65 | - | 0.51 | -0.07 | (0.13) | 0.6170 | -0.33 | - | 0.19 | -4.41 | -0.53 | (0.19) | 0.0054 | -0.90 | - | -0.16 | -0.10 | (0.08) | 0.1663 | -0.25 | - | 0.04 | -7.10 |
| A bit milder than expected | -0.02 | (0.30) | 0.9419 | -0.61 | - | 0.56 | 0.00 | (0.13) | 0.9823 | -0.26 | - | 0.26 | 0.08 | -0.04 | (0.21) | 0.8476 | -0.44 | - | 0.36 | 0.01 | (0.08) | 0.9349 | -0.14 | - | 0.15 | -0.05 |
| As severe/mild as expected | . |  |  |  |  |  | . |  |  |  |  |  | . | . |  |  |  |  |  | . |  |  |  |  |  | . |
| A bit more severe than expected | -0.02 | (0.33) | 0.9537 | -0.66 | - | 0.62 | -0.05 | (0.15) | 0.7513 | -0.35 | - | 0.25 | -3.08 | 0.48 | (0.23) | 0.0384 | 0.03 | - | 0.94 | 0.14 | (0.08) | 0.0757 | -0.01 | - | 0.29 | 8.56 |
| More severe than expected | 0.03 | (0.42) | 0.9502 | -0.80 | - | 0.85 | -0.06 | (0.19) | 0.7694 | -0.43 | - | 0.32 | -3.29 | 0.44 | (0.26) | 0.0989 | -0.08 | - | 0.95 | 0.18 | (0.09) | 0.0475 | 0.00 | - | 0.36 | 10.23 |
| Mixed, i.e. half more severe, half milder than expected | -0.64 | (0.36) | 0.0756 | -1.34 | - | 0.07 | -0.01 | (0.19) | 0.9392 | -0.40 | - | 0.37 | -4.50 | -0.23 | (0.29) | 0.4351 | -0.81 | - | 0.35 | -0.19 | (0.11) | 0.0934 | -0.41 | - | 0.03 | -7.54 |
| No HIS | . |  |  |  |  |  | . |  |  |  |  |  | . | . |  |  |  |  |  | . |  |  |  |  |  | . |
| Female | 0.08 | (0.14) | 0.5648 | -0.20 | - | 0.37 | -0.03 | (0.07) | 0.6794 | -0.18 | - | 0.12 | -1.56 | 0.27 | (0.11) | 0.0119 | 0.06 | - | 0.47 | 0.01 | (0.04) | 0.8644 | -0.08 | - | 0.09 | 2.08 |
| Age |  |  |  |  |  |  |  |  |  |  |  |  |  |  |  |  |  |  |  |  |  |  |  |  |  |  |
| 18-24 years old | -0.18 | (0.43) | 0.6790 | -1.01 | - | 0.66 | 0.66 | (0.23) | 0.0050 | 0.20 | - | 1.12 | 56.16 | -0.26 | (0.24) | 0.2760 | -0.74 | - | 0.21 | -0.16 | (0.10) | 0.1242 | -0.36 | - | 0.04 | -7.79 |
| 25-34 years old | -0.51 | (0.30) | 0.0881 | -1.09 | - | 0.08 | 0.01 | (0.15) | 0.9686 | -0.30 | - | 0.31 | -1.67 | -0.46 | (0.21) | 0.0294 | -0.87 | - | -0.05 | -0.10 | (0.09) | 0.2990 | -0.28 | - | 0.09 | -6.91 |
| 35-44 years old | -0.37 | (0.26) | 0.1575 | -0.87 | - | 0.14 | 0.03 | (0.13) | 0.8311 | -0.22 | - | 0.28 | 0.28 | -0.52 | (0.21) | 0.0120 | -0.92 | - | -0.11 | -0.10 | (0.09) | 0.2488 | -0.28 | - | 0.07 | -7.59 |
| 45-54 years old | -1.10 | (0.20) | <.0001 | -1.49 | - | -0.71 | -0.01 | (0.11) | 0.9106 | -0.23 | - | 0.20 | -6.17 | -0.39 | (0.21) | 0.0639 | -0.80 | - | 0.02 | -0.08 | (0.09) | 0.3588 | -0.27 | - | 0.10 | -5.98 |
| 55-64 years old | -0.39 | (0.21) | 0.0577 | -0.80 | - | 0.01 | 0.02 | (0.10) | 0.8290 | -0.17 | - | 0.22 | -0.19 | 0.09 | (0.23) | 0.6955 | -0.36 | - | 0.54 | -0.24 | (0.10) | 0.0115 | -0.43 | - | -0.05 | -8.60 |
| 65 years old and older | . |  |  |  |  |  | . |  |  |  |  |  | . | . |  |  |  |  |  | . |  |  |  |  |  | . |
| Education level |  |  |  |  |  |  |  |  |  |  |  |  |  |  |  |  |  |  |  |  |  |  |  |  |  |  |
| High | 0.12 | (0.22) | 0.5726 | -0.31 | - | 0.55 | 0.20 | (0.12) | 0.0969 | -0.04 | - | 0.43 | 11.93 | 0.12 | (0.16) | 0.4409 | -0.19 | - | 0.44 | 0.17 | (0.07) | 0.0183 | 0.03 | - | 0.31 | 6.68 |
| Middle | -0.14 | (0.21) | 0.5156 | -0.55 | - | 0.28 | 0.23 | (0.12) | 0.0527 | 0.00 | - | 0.47 | 13.03 | -0.07 | (0.16) | 0.6475 | -0.38 | - | 0.24 | 0.10 | (0.07) | 0.1831 | -0.05 | - | 0.24 | 2.65 |
| Low | . |  |  |  |  |  | . |  |  |  |  |  | . | . |  |  |  |  |  | . |  |  |  |  |  | . |
| Level of making ends meet |  |  |  |  |  |  |  |  |  |  |  |  |  |  |  |  |  |  |  |  |  |  |  |  |  |  |
| Easily | 2.56 | (0.32) | <.0001 | 1.93 | - | 3.19 | 0.50 | (0.21) | 0.0149 | 0.10 | - | 0.91 | 49.80 | 1.61 | (0.24) | <.0001 | 1.14 | - | 2.07 | 0.37 | (0.10) | 0.0003 | 0.17 | - | 0.57 | 25.07 |
| Fairly easy | 2.13 | (0.25) | <.0001 | 1.65 | - | 2.61 | 0.16 | (0.19) | 0.4109 | -0.22 | - | 0.54 | 22.40 | 1.12 | (0.18) | <.0001 | 0.76 | - | 1.48 | 0.17 | (0.09) | 0.0498 | 0.00 | - | 0.35 | 13.31 |
| With some difficulty | 0.96 | (0.22) | <.0001 | 0.54 | - | 1.39 | 0.04 | (0.19) | 0.8214 | -0.34 | - | 0.42 | 10.27 | 0.53 | (0.18) | 0.0024 | 0.19 | - | 0.88 | 0.10 | (0.09) | 0.2712 | -0.08 | - | 0.27 | 6.57 |
| With great difficulty | . |  |  |  |  |  | . |  |  |  |  |  | . | . |  |  |  |  |  | . |  |  |  |  |  | . |
| Country |  |  |  |  |  |  |  |  |  |  |  |  |  |  |  |  |  |  |  |  |  |  |  |  |  |  |
| Denmark | 0.17 | (0.27) | 0.5430 | -0.37 | - | 0.71 | -0.03 | (0.14) | 0.8314 | -0.30 | - | 0.24 | -1.08 | -0.24 | (0.23) | 0.2971 | -0.70 | - | 0.21 | -0.20 | (0.11) | 0.0595 | -0.41 | - | 0.01 | -8.06 |
| Spain | -0.35 | (0.30) | 0.2328 | -0.94 | - | 0.23 | -0.27 | (0.16) | 0.0924 | -0.59 | - | 0.05 | -16.86 | -0.30 | (0.20) | 0.1379 | -0.70 | - | 0.10 | -0.67 | (0.09) | <.0001 | -0.84 | - | -0.50 | -20.94 |
| France | 0.06 | (0.25) | 0.8179 | -0.44 | - | 0.55 | -0.50 | (0.14) | 0.0005 | -0.77 | - | -0.22 | -25.99 | -0.35 | (0.21) | 0.0899 | -0.75 | - | 0.05 | -0.61 | (0.10) | <.0001 | -0.80 | - | -0.43 | -19.15 |
| Italy | 0.38 | (0.32) | 0.2292 | -0.24 | - | 1.00 | -0.31 | (0.16) | 0.0471 | -0.61 | - | 0.00 | -15.96 | 0.40 | (0.22) | 0.0631 | -0.02 | - | 0.82 | -0.47 | (0.09) | <.0001 | -0.64 | - | -0.30 | -12.51 |
| Netherlands | -0.09 | (0.29) | 0.7560 | -0.67 | - | 0.48 | 0.09 | (0.15) | 0.5796 | -0.22 | - | 0.39 | 5.09 | -0.39 | (0.21) | 0.0564 | -0.80 | - | 0.01 | -0.26 | (0.09) | 0.0051 | -0.44 | - | -0.08 | -10.77 |
| Portugal | 1.09 | (0.48) | 0.0221 | 0.16 | - | 2.03 | -0.87 | (0.17) | <.0001 | -1.20 | - | -0.53 | -36.42 | 1.13 | (0.23) | <.0001 | 0.68 | - | 1.58 | -0.89 | (0.09) | <.0001 | -1.06 | - | -0.73 | -20.83 |
| United Kingdom | -0.22 | (0.25) | 0.3636 | -0.71 | - | 0.26 | -0.13 | (0.13) | 0.3165 | -0.39 | - | 0.13 | -8.96 | -0.45 | (0.24) | 0.0601 | -0.92 | - | 0.02 | -0.27 | (0.11) | 0.0130 | -0.49 | - | -0.06 | -11.31 |
| Germany |  |  |  |  |  |  |  |  |  |  |  |  |  |  |  |  |  |  |  |  |  |  |  |  |  |  |
| Health Problematization Index | -0.02 | (0.02) | 0.3497 | -0.06 | - | 0.02 | 0.01 | (0.01) | 0.5013 | -0.02 | - | 0.03 | 0.45 | 0.01 | (0.02) | 0.3895 | -0.02 | - | 0.05 | 0.01 | (0.01) | 0.1040 | 0.00 | - | 0.02 | 0.50 |
| Risk to own health from COVID-19 |  |  |  |  |  |  |  |  |  |  |  |  |  |  |  |  |  |  |  |  |  |  |  |  |  |  |
| No risk at all | -1.23 | (0.34) | 0.0003 | -1.91 | - | -0.56 | -0.31 | (0.20) | 0.1253 | -0.70 | - | 0.09 | -24.68 | -1.48 | (0.25) | <.0001 | -1.96 | - | -0.99 | -0.26 | (0.11) | 0.0219 | -0.48 | - | -0.04 | -18.82 |
| Little risk | -0.62 | (0.27) | 0.0239 | -1.15 | - | -0.08 | -0.07 | (0.14) | 0.6291 | -0.34 | - | 0.20 | -7.57 | -0.70 | (0.21) | 0.0008 | -1.11 | - | -0.29 | -0.12 | (0.08) | 0.1571 | -0.28 | - | 0.04 | -9.20 |
| Moderate risk | -0.36 | (0.25) | 0.1395 | -0.84 | - | 0.12 | -0.19 | (0.12) | 0.1115 | -0.43 | - | 0.04 | -14.06 | -0.27 | (0.20) | 0.1753 | -0.66 | - | 0.12 | -0.06 | (0.08) | 0.4045 | -0.21 | - | 0.08 | -4.30 |
| High risk | -0.23 | (0.25) | 0.3683 | -0.72 | - | 0.27 | -0.06 | (0.13) | 0.6217 | -0.31 | - | 0.18 | -5.27 | -0.11 | (0.22) | 0.5942 | -0.54 | - | 0.31 | -0.02 | (0.08) | 0.8058 | -0.18 | - | 0.14 | -1.59 |
| Very high risk | . |  |  |  |  |  | . |  |  |  |  |  | . | . |  |  |  |  |  | . |  |  |  |  |  | . |
| Risk aversion |  |  |  |  |  |  |  |  |  |  |  |  |  |  |  |  |  |  |  |  |  |  |  |  |  |  |
| Very low | 0.26 | (0.25) | 0.2990 | -0.23 | - | 0.75 | -0.11 | (0.14) | 0.4165 | -0.38 | - | 0.16 | -5.89 | -0.37 | (0.18) | 0.0371 | -0.71 | - | -0.02 | -0.34 | (0.07) | <.0001 | -0.47 | - | -0.21 | -15.87 |
| Low | 0.46 | (0.27) | 0.0919 | -0.07 | - | 0.99 | 0.01 | (0.14) | 0.9633 | -0.26 | - | 0.28 | 2.65 | -0.68 | (0.20) | 0.0005 | -1.07 | - | -0.30 | -0.37 | (0.08) | <.0001 | -0.54 | - | -0.21 | -18.86 |
| Moderately | 0.11 | (0.18) | 0.5557 | -0.25 | - | 0.47 | -0.15 | (0.10) | 0.1434 | -0.35 | - | 0.05 | -8.85 | -0.79 | (0.13) | <.0001 | -1.05 | - | -0.54 | -0.43 | (0.05) | <.0001 | -0.54 | - | -0.33 | -21.15 |
| High | . |  |  |  |  |  | . |  |  |  |  |  | . | . |  |  |  |  |  | . |  |  |  |  |  | . |
| Vaccination status | --- |  |  |  |  |  |  |  |  |  |  |  |  |  |  |  |  |  |  |  |  |  |  |  |  |  |
| No |  |  |  |  |  |  |  |  |  |  |  |  |  | . |  |  |  |  |  | . |  |  |  |  |  | . |
| Not yet, but I intend to |  |  |  |  |  |  |  |  |  |  |  |  |  | 1.99 | (0.29) | <.0001 | 1.41 | - | 2.56 | -0.25 | (0.20) | 0.1964 | -0.64 | - | 0.13 | 14.79 |
| Yes, the first shot |  |  |  |  |  |  |  |  |  |  |  |  |  | 3.12 | (0.26) | <.0001 | 2.62 | - | 3.63 | -0.24 | (0.16) | 0.1216 | -0.54 | - | 0.06 | 25.55 |
| Yes, both shots |  |  |  |  |  |  |  |  |  |  |  |  |  | 3.21 | (0.19) | <.0001 | 2.84 | - | 3.57 | -0.23 | (0.14) | 0.0963 | -0.50 | - | 0.04 | 26.64 |
| Yes, three shots (booster) |  |  |  |  |  |  |  |  |  |  |  |  |  | --- |  |  |  |  |  | --- |  |  |  |  |  |  |
| Peers' vaccination status |  |  |  |  |  |  |  |  |  |  |  |  |  |  |  |  |  |  |  |  |  |  |  |  |  |  |
| Most | 1.39 | (0.57) | 0.0152 | 0.27 | - | 2.51 | -0.09 | (0.55) | 0.8688 | -1.17 | - | 0.99 | 4.47 | 0.24 | (0.29) | 0.4018 | -0.32 | - | 0.80 | -0.11 | (0.14) | 0.4436 | -0.39 | - | 0.17 | -2.36 |
| About half | 1.06 | (0.63) | 0.0922 | -0.17 | - | 2.30 | 0.54 | (0.58) | 0.3571 | -0.60 | - | 1.68 | 53.27 | -0.55 | (0.31) | 0.0776 | -1.16 | - | 0.06 | -0.01 | (0.15) | 0.9536 | -0.31 | - | 0.29 | -4.62 |
| Just a few | 1.44 | (0.64) | 0.0235 | 0.19 | - | 2.68 | 0.27 | (0.57) | 0.6357 | -0.85 | - | 1.39 | 30.65 | -0.34 | (0.30) | 0.2668 | -0.93 | - | 0.26 | 0.12 | (0.15) | 0.3998 | -0.17 | - | 0.42 | 2.00 |
| None | . |  |  |  |  |  | . |  |  |  |  |  | . | . |  |  |  |  |  | . |  |  |  |  |  | . |
|  |  |  |  |  |  |  |  |  |  |  |  |  |  |  |  |  |  |  |  |  |  |  |  |  |  |  |
| AME - average marginal effect, Est. - estimate, No. - number, SE - standard error | | | | | | |  |  |  |  |  |  |  |  |  |  |  |  |  |  |  |  |  |  |  |  |
